# The Action of Quinine on the Fœtus

**Published:** 1886-07-20

**Authors:** 


**The Action of Quinine on the Fœtus.**—Vadenuke, in *N. Y. Med. Journal*, arrives at the following conclusions: 1. Quinine taken by the mother passes into the system of the fœtus in the proportion of about one-ninth of the whole amount. 2. The largest amount is contained in the fœtus at the end of two hours. 3. The fœtus eliminates it in a little more than forty-eight hours, and the neonatus in seventy-two hours. 4. Large therapeutic doses given to the mother do no harm to the fœtus. 5. The same is true of large doses repeated every forty-eight hours. 6. Quinine is not an abortifacent. 7. It may ward off abortion or premature labor when the mother is under the influence of fever, especially of malarial orgin.—*Medical Times.*

